# Genomic Features and Clinical Implications of Intraductal Carcinoma of the Prostate

**DOI:** 10.3390/ijms222313125

**Published:** 2021-12-04

**Authors:** Minyong Kang, Hyunwoo Lee, Sun-Ju Byeon, Ghee Young Kwon, Seong Soo Jeon

**Affiliations:** 1 Department of Urology, Samsung Medical Center, Sungkyunkwan University School of Medicine, 81 Irwon-ro, Gangnam-gu, Seoul 06351, Korea; m79.kang@skku.edu; 2 Samsung Genome Institute, Samsung Medical Center, Seoul 06351, Korea; 3 Department of Health Sciences and Technology, SAIHST, Sungkyunkwan University, Seoul 06351, Korea; 4 Department of Digital Health, SAIHST, Sungkyunkwan University, Seoul 06351, Korea; 5 Department of Pathology and Translational Genomics, Samsung Medical Center, Sungkyunkwan University School of Medicine, Seoul 06351, Korea; hwpatho.lee@samsung.com; 6 Department of Pathology, Hallym University Dongtan Sacred Heart Hospital, Hwaseong-si 18450, Korea; byeon.sunju@welovedoctor.com

**Keywords:** prostate cancer, intraductal carcinoma, genomic feature, clinical implication

## Abstract

Intraductal carcinoma of the prostate (IDC-P) is a rare and unique form of aggressive prostate carcinoma, which is characterized by an expansile proliferation of malignant prostatic epithelial cells within prostatic ducts or acini and the preservation of basal cell layers around the involved glands. The vast majority of IDC-P tumors result from adjacent high-grade invasive cancer via the retrograde spreading of tumor cells into normal prostatic ducts or acini. A subset of IDC-P tumors is rarely derived from the de novo intraductal proliferation of premalignant cells. The presence of IDC-P in biopsy or surgical specimens is significantly associated with aggressive pathologic features, such as high Gleason grade, large tumor volume, and advanced tumor stage, and with poor clinical courses, including earlier biochemical recurrence, distant metastasis, and worse survival outcomes. These architectural and behavioral features of IDC-P may be driven by specific molecular properties. Notably, IDC-P possesses distinct genomic profiles, including higher rates of *TMPRSS2–ERG* gene fusions and *PTEN* loss, increased percentage of genomic instability, and higher prevalence of germline *BRCA2* mutations. Considering that IDC-P tumors are usually resistant to conventional therapies for prostate cancer, further studies should be performed to develop optimal therapeutic strategies based on distinct genomic features, such as treatment with immune checkpoint blockades or poly (adenosine diphosphate–ribose) polymerase inhibitors for patients harboring increased genomic instability or *BRCA2* mutations, as well as genetic counseling with genetic testing. Patient-derived xenografts and tumor organoid models can be the promising in vitro platforms for investigating the molecular features of IDC-P tumor.

## 1. Introduction

Intraductal carcinoma of the prostate (IDC-P) is a unique and aggressive morphologic variant of prostate adenocarcinoma that is usually associated with unfavorable pathologic features such as advanced-stage, high-grade, and large-volume tumors [1]. IDC-P has two morphological features, which are characterized by (i) a lumen-spanning growth of atypical prostate cancer cells within pre-existing prostatic ducts/acini, and (ii) at least a partial preservation of basal cell layers [2].

Although IDC-P prevalence varied across studies, Porter et al. [3] found that IDC-P prevalence increased from 2.1% in low-risk patients to 23.1%, 36.7%, and 56.0% in moderate-risk patients, high-risk patients, and patients with metastatic diseases, respectively, after performing a systematic review of 38 prostate cancer cohorts. Moreover, the presence of IDC-P in biopsy or radical prostatectomy specimens is strongly associated with early recurrence after initial treatment, as well as reduced therapeutic response to androgen-deprivation therapy (ADT) or taxane chemotherapy in metastatic diseases [4]. In this context, IDC-P was categorized as a biologically and pathologically distinct entity from the acinar adenocarcinoma of the prostate by the 2016 World Health Organization Classification of Tumors of the Prostate Gland [5].

Despite growing evidence on the association between IDC-P and unfavorable clinical scenarios, information on optimal treatment strategies remains largely unclear. To develop effective therapeutics that improve the clinical outcomes of patients with IDC-P histology, the molecular pathogenesis should be precisely elucidated. Although knowledge on the genetic features of prostate adenocarcinoma has recently progressed, little is known about the genetics of IDC-P. The aim of this review is to summarize the current understanding of the genomic landscapes of IDC-P and discuss its clinical implications.

## 2. Genomic Alterations of IDC-P

IDC-P and acinar adenocarcinoma of the prostate have highly conserved genomic profiles and are clonally related, thus indicating that they may originate from the same progenitor cells [6]. Furthermore, the identification of reliable genomic markers for IDC-P remains a challenging issue. We comprehensively review the key genomic features of IDC-P tumors, particularly somatic mutations, genomic instability, and DNA repair gene mutations. Table 1 summarizes the clinicopathological and genomic features of IDC-P.

### 2.1. Somatic Mutation

IDC-P shares immunohistochemical features, such as positive staining of prostate-specific antigen and alpha-methylacyl-CoA racemase, with high-grade prostatic intraepithelial neoplasia (HGPIN), which is known as precancerous lesion of prostate cancer (PCa) [30]. Additionally, some IDC-P tumors have morphologic features that overlap with HGPIN, including loose cribriform and micropapillary patterns [31]. However, the number and frequency of molecular changes in IDC-P are much higher than in HGPIN, indicating that they are biologically distinct lesions [31]. For instance, *ERG* rearrangement was absent in isolated cribriform HGPIN, whereas it was observed in majority of IDC-P tumors [12]. Moreover, loss of heterozygosity (LOH) of both *TP53* and *RB1* genes was more frequently detected in IDC-P (52%) compared to HGPIN (19%) [13]. Therefore, molecular features of IDC-P clearly distinguish it from HGPIN.

*TMPRSS2–ERG* gene fusion is the most common recurrent genetic alteration in prostatic adenocarcinoma and has been identified in approximately 50% of cases [14]. In cases with IDC-P components, *ERG* gene rearrangements were detected in 75% by fluorescence in situ hybridization assays [12]. In addition to *ERG* gene fusions, the loss of *RB1*, *TP53*, and *PTEN*, as well as *MYC* amplification, is highly detected in tumors with IDC-P [15]. Exome sequencing data from The Cancer Genome Atlas (TCGA) showed that patients with IDC-P components had higher rates of point mutations in *FOXA1* (15% vs. 5%), *TP53* (19% vs. 10%), and *SPOP* (19% vs. 10%) than in those without IDC-P [32]. In addition, IDC-P carriers had a unique AR pathway aberration, such as enrichment of *NCOR2* mutation, compared with individuals with pure prostate adenocarcinoma [20].

Interestingly, Han et al. [12] reported that 100% of IDC-P cases showed concordance of ERG fusion status with adjacent invasive carcinoma, thus suggesting that a clonal relationship exists between IDC-P and adenocarcinoma. Additionally, 100% of cases with positive ERG expression by immunohistochemical staining in the IDC-P component also had a positive ERG expression in the adjacent invasive prostatic adenocarcinoma and vice versa [33]. The deletion or mutation of the *PTEN* tumor suppressor gene is another common somatic genomic alteration in prostate cancer and is usually associated with advanced-stage disease [34]. In a study assessing *PTEN* loss by using immunohistochemical assays, *PTEN* loss was strongly associated with IDC-P (69% of total samples with *PTEN* loss vs. 12% of *PTEN* intact cases), and IDC-P had the highest relative risk (4.99; 95% confidence interval (CI) = 3.451–7.223) for the loss of *PTEN* [15]. Moreover, cytoplasmic *PTEN* expression status was highly concordant (>95%) between IDC-P-positive lesions and concurrent invasive adenocarcinoma [35]. These data indicate a shared clonal relationship between IDC-P components and adjacent high-grade invasive prostatic adenocarcinoma and strongly suggest that the growth of IDC-P is associated with retrograde spread.

**Figure 1:**
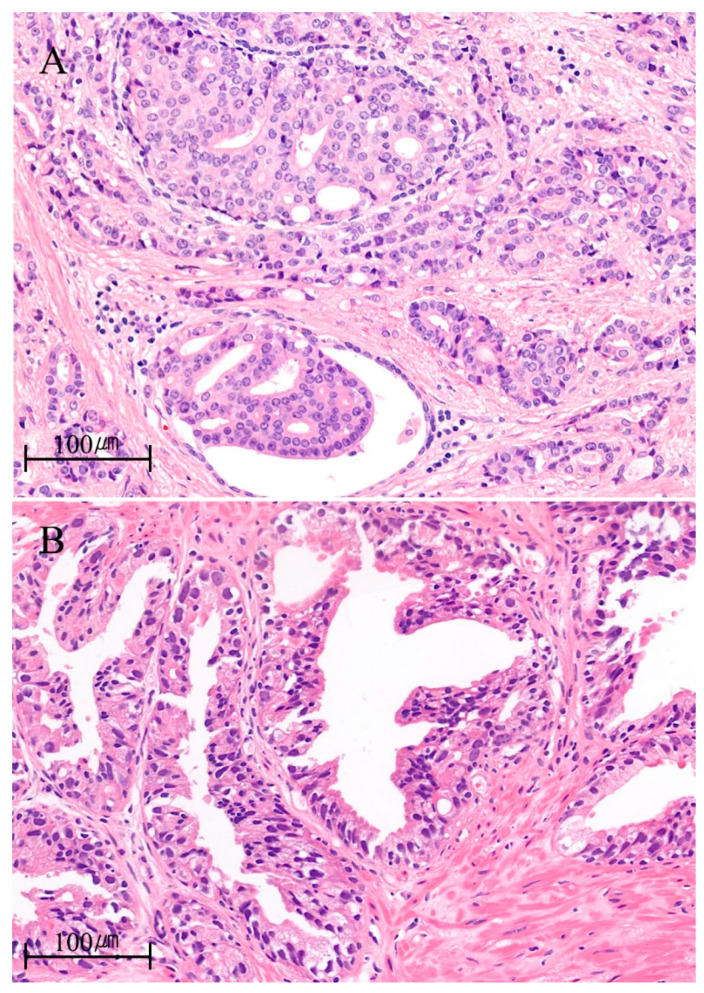
Representative images of hematoxylin and eosin staining for (**A**) classic and (**B**) isolated forms of intraductal carcinoma of the prostate (IDC-P) from the cases of the authors′ institution. (**A**) Cribriform tumor spreads into a non-neoplastic gland lined by a layer of basal cells in a 50 year old patient with pT3b prostate cancer. Initial PSA was 29.22 ng/mL. The Gleason score of invasive adenocarcinoma was 4 + 3 = 7/10. (**B**) Intraductal proliferation shows epithelial atypia surpassing that of high-grade prostatic intraepithelial neoplasia (HGPIN) not accompanied by invasive adenocarcinoma elsewhere in an 80 year old patient. Initial PSA was 7.09 ng/mL.

However, some data showed that IDC-P was an isolated finding in radical prostatectomy specimens as a separate de novo form adjoining high-grade invasive carcinoma [24,36,37]. Miyai et al. [38] reported that 90% of tumors (*n* = 141/155) were categorized as a regular type of IDC-P in the absence of invasive carcinoma, and 10% of tumors (*n* = 14/155) were precursor-like IDC-P tumors without an associated invasive component. In particular, approximately 2% of tumors (*n* = 3/155) were found to be pure IDC-P tumors in the absence of any invasive lesion and were located more than 3 mm from invasive tumor regions in surgical specimens [38]. Patients with precursor-like IDC-P had better clinicopathological features and longer biochemical recurrence (BCR)-free survival than those with classical IDC-P [38]. Consistent with these findings, Khani et al. [24] demonstrated that IDC-P without invasive tumors had good clinical prognosis and distinct molecular features, such as a striking number (57%) of enrichments in oncogenic driver mutations in MAPK/PI3K pathway genes in targeted sequencing, which are rare in conventional prostate adenocarcinoma. These data suggest that a small subset of IDC-P can occur as an isolated precancerous lesion with the de novo intraductal outgrowth of tumors with different clinical behaviors and genomic characteristics compared with those via the retrograde spreading of cancer cells. Figure 1 shows the representative cases of classical intraductal carcinoma of the IDC-P via the retrograde spreading of tumor cells, as well as an isolated form of IDC-P.

### 2.2. Genomic Instability

Several studies have found that genomic instability is another key adverse molecular index of IDC-P. Chua et al. [10] reported that the IDC-P component and cribriform architecture (CA) were lethal sub-pathologies of PCa, which predicted an increased risk of BCR and distant metastasis. They further revealed that IDC-P and CA were significantly associated with a “nimbosus” phenomenon of prostate cancer, which is characterized by an increased percentage of genome alteration (median 7.2 vs. 3.0%), hypoxia (64.0% vs. 45.5%), and long noncoding RNA *SChLAP1* (SWI/SNF complex antagonist associated with prostate cancer 1) abundance (>3-fold higher expression) [10]. Consistent with these findings, the multiparametric magnetic resonance imaging-visible tumors had a higher prevalence of IDC-P and CA pathology with the hallmarks of “nimbosus”, including higher mutation density, increased genomic instability, and enrichment of *SChLAP1* expression [17]. Interestingly, higher expression of *SChLAP1* was strongly associated with higher Gleason score and higher chance of developing BCR, metastasis, and lethal disease [18,19]. Furthermore, the association with poor prognosis is intensified when tumor hypoxia is incorporated with other prognostic factors, such as increased PGA and *PTEN* loss [16].

Böttcher et al. [32] hypothesized that IDC-P and CA are specific histomorphological subgroups that harbor unique genomic alterations associated with aggressive clinical behavior, including chromothripsis and copy number alterations. They reviewed whole-slide images of the TCGA database and the radical prostatectomy datasets of the Canadian Prostate Cancer Genome Network. The authors found that the presence of IDC-P and CA histology was significantly associated with increased genomic instability, and it affected specific genomic regions: chromosomal deletions of 3p13, 6q15, 8p21–23, 10q23, 13q14, 16q21–24, and 18q21–23; amplification of chromosome 8q24 [32]. Additionally, chromosomal deletions and amplifications included several genes associated with aggressive PCa, including *PTEN, TP53,* and *RB1* loss and *MYC* amplification [32].

Consistent with these findings, loss of heterozygosity of *TP53* and *RB1* genes was more frequently found in IDC-P than in other Gleason grade 3 and 4 patterns or high-grade dysplasia [13]. Williams et al. [39] performed a systematic review using public genomic datasets of PCa, followed by a comprehensive meta-analysis of 662 tumor samples, to derive a consensus map of recurrent somatic copy number alterations (CNAs). Notably, the prevalence of most frequent CNAs in 161 advanced tumors was similar with several of the CNAs enriched in tumors with IDC-P and CA histology, such as *PTEN* (10q) and *NKX3-1* (8p) [39]. More interestingly, a recent study by Chen et al. [40] characterized the circular RNA transcriptional landscape of 144 localized prostate cancers using ultradeep total RNA sequencing; they found a unique linear transcriptomic subtype (P1) tumor showing high global genomic instability and a strong association with aggressive IDC-P and CA sub-histology. For instance, 63% of tumors in the P1 subtype had IDC-P and CA morphology compared with 20–40% of those in other linear transcriptomic subtypes [40].

These findings on the genomic instability of IDC-P tumors shed light on the strong relationship of this specific subset of prostate cancer with molecular tumor progression and disease aggressiveness.

### 2.3. DNA Repair Gene Mutation

Patients with germline mutations in homologous DNA recombination repair (HRR) genes, such as *BRCA1, BRCA2, ATM, PALB2*, and *CHEK2*, are at a higher risk of prostate cancer development and usually have worse prognosis [21]. The prevalence of germline DNA repair genes was approximately 12% among patients with metastatic prostate cancer, which was significantly higher than that among patients with localized prostate cancer [22]. Particularly, deleterious defects in the HRR pathways were found in at least 20–25% of men with metastatic castration-resistant prostate cancer (mCRPC). Among 150 patients with recurrent or metastatic prostate cancer, the HRR mutation rates were higher in patients harboring IDC-P histology than in those without this histology (40% vs. 9%, respectively), and patients with defects in HRR genes had a higher prevalence of intraductal histology than those without these mutations (48% vs. 12%, respectively) [23].

In cases of *BRCA2* germline mutations, the prevalence of an IDC-P-positive tumor was markedly higher than those without genetic risk factors, such as familial history (42% vs. 9%) [11]. Interestingly, patient-derived tumor xenografts obtained from three patients with *BRCA2* germline mutations showed a higher rate of IDC-P components than samples derived from sporadic cases [11]. Concomitant IDC-P tumor was also significantly associated with poor progression-free survival (PFS) and overall survival (OS) [11].

Germline *BRCA2*-mutant prostatic tumors harbor various dysregulation of signaling pathways related to aggressive tumor biology, including amplification of the WNT pathway modulator *MED12L* [41]. In a study by Taylor et al. [5], the genomes and methylomes were comprehensively analyzed in 14 patients with prostate cancer with germline *BRCA2* mutation (*BRCA2* mutant). Of note, the genomic and epigenomic dysregulation of the WNT/β-catenin pathway modulator *MED12/MED12L* axis was enriched in *BRCA2*-mutant tumors with an IDC component, but was not enriched in sporadic tumors with IDC-P [41]. These data suggest that WNT pathway modulation plays a key role in the etiology of aggressive *BRCA2*-mutant tumors with concurrent IDC-P histology. More importantly, they identified that IDC and adjacent non-IDC components shared a common precursor before multiple branching into subspecies, thus indicating that the initiation and progression of IDC-P may be driven by subsequent genomic and epigenomic dysregulation during early tumorigenesis [41]. Taken together, the mechanisms of IDC-P development might be clearly different between *BRCA2*-mutant and sporadic IDC-P-positive tumors [41].

Velho et al. [23] analyzed 150 men with recurrent or metastatic prostate cancer who underwent germline genetic testing and found that 24% of patients (*n* = 5/21) with germline mutations had intraductal histology, whereas only 9.3% of cases (*n* = 12/129) had intraductal histology in those without germline mutations. More interestingly, deleterious germline DNA repair gene mutations, including *BRCA2, ATM, CHEK2,* and *BRCA1*, were detected in 40% of patients (*n* = 10/25) with IDC-P compared with only 9% (*n* = 11/125) of those without IDC-P [23]. Zhao et al. [20] performed a targeted sequencing of circulating cell-free DNA obtained from 164 IDC-P carriers and 84 IDC-P noncarriers of prostate adenocarcinoma. They found that nearly one-third of patients with metastatic prostate cancer with IDC-P histology had DNA repair defects [20]. More than one-third (35.6%) of IDC-P-positive cases had alterations of DNA repair pathways in the CRPC cohorts, and this finding was approximately 10% higher than the results from previous studies [20]. More interestingly, pathogenic germline mutations in DNA repair genes, including *BRCA2* and *CDK12*, were more frequently detected in patients with IDC-P than in those without IDC-P. For instance, the prevalence of germline *BRCA2* mutations was significantly higher in patients with IDC-P than in those with adenocarcinoma of the prostate (8.7% vs. 0%, respectively) [20]. For IDC-P carriers at CRPC status, patients with *BRCA2* mutation showed shorter PSA-PFS after first-line abiraterone acetate administration (median 9.1 versus 11.9 months).

These results suggest that various germline mutations in DNA repair genes may contribute to increased genomic instability and consequently influence IDC-P development. Therefore, we believe that the presence of IDC-P histology can be an early indicator of recommendations for genetic counseling and germline genetic testing for DNA repair gene mutations. The guidelines of the National Comprehensive Cancer Network and the Philadelphia Prostate Cancer Consensus Conference also recommend genetic testing and counseling for all men with IDC-P and cribriform histology-positive prostate cancer, regardless of family history or risk classification [42].

## 3. Genomic Features and Its Clinical Implications

Kweldam et al. [8] examined the diagnostic biopsies of 1031 men with prostate cancer from the European Randomized Study of Screening for Prostate Cancer (1993–2000) and found that the presence of IDC-P was significantly associated with worse disease-specific survival in the multivariate analysis regardless of Gleason score (hazard ratio (HR) = 2.6; 95% CI = 1.4–4.8). They also demonstrated that the presence of any volume of IDC-P (focal vs. extensive) was a highly significant prognostic factor for the BCR-free rate (HR = 2.98, 95% CI = 1.68–5.28), thus indicating that the presence of IDC-P itself is a worse prognostic factor [8,9]. More interestingly, previous studies reported that the IDC-P component persisted after ADT, thus suggesting the inherent resistance of IDC-P to conventional systemic therapy in prostate cancer [25,26]. However, Kato et al. [27] reported that some IDC-P-positive cases responded well to ADT and showed the disappearance of IDC-P components in RP specimens from 145 men with high-risk prostate cancer with good survival outcomes. Therefore, there might be heterogeneous responses to ADT in tumors with IDC-P histology, and underlying molecular features related to responsiveness or resistance should be discovered to improve treatment outcomes.

Yamamoto et al. [28] analyzed 79 mCRPC patients and found that docetaxel treatment significantly improved the survival outcomes in cases with IDC-P component compared to those without chemotherapy treatment (median cancer-specific survival = 20.5 months vs. 7 months, respectively). More importantly, the presence of IDC-P histology in needle biopsies at the time of initial diagnosis and docetaxel treatment were identified as significant prognostic factors for survival outcomes in multivariate analysis [28]. They also performed propensity score matching in 234 patients with mCRPC who received docetaxel or AR axis targeting (ARAT) agents (abiraterone acetate or enzalutamide) as first-line systemic therapy [29]. Among patients with IDC-P tumors, OS was significantly longer in patients receiving ARAT agents than in those treated with docetaxel (HR = 0.48; 95% CI = 0.26–0.86) [29]. Furthermore, multivariate analysis revealed that the presence of the IDC-P component was identified as a prognostic factor for OS, in addition to the duration of ADT, presence of visceral metastasis, and treatment with ARAT agents as first-line systemic therapy [29]. Although no association was detected between genomic alterations and PSA response, *TP53* alteration was associated with rapid progression to castration-resistant status, and *BRCA2* mutation was related to a short PSA-PFS in mCRPC patients harboring IDC-P components receiving first-line abiraterone treatment [20].

These findings may offer clues to proper therapeutic options for patients with IDC-P, particularly patients with mCRPC. Recently, several phase 3 trials, including CHARRTED, LATITUDE, and ENZAMET trials, confirmed that the use of early docetaxel and ARAT agents was significantly more effective in patients with metastatic hormone-sensitive prostate cancer (mHSPC) than in those with mCRPC, particularly in high-volume or high-risk patients [43,44,45]. In this regard, a paradigm shift has occurred in clinical practice for metastatic prostate cancer; therefore, genomic profiling and its prognostic effect should be investigated in mHSPC patients harboring the IDC-P histology to define the effective agents and optimal timing of docetaxel or ARAT treatments in these patients.

The association between genomic instability and neoantigen creation is pertinent to treatment using immune checkpoint blockades (ICBs), such as pembrolizumab (anti-PD-1 immune checkpoint inhibitor), which has been approved for metastatic tumors with high microsatellite instability/deficient mismatch repair (MMR) [46]. Le et al. [47] conducted a phase 2 trial to determine the efficacy of pembrolizumab in 41 patients with metastatic colorectal cancers according to the presence of MMR deficiency. They found that objective response rate and progression-free survival rate at 20 weeks were 40% and 78%, respectively, for the MMR-deficient population, but 0% and 11%, respectively, for the MMR-proficient subset [47]. This research group expanded the previous study to investigate the clinical activity of pembrolizumab in patients harboring 12 different types of advanced cancers with MMR deficiency [48]. Notably, the objective response rate was 52% in patients with colorectal cancers and 54% in patients with other type of cancers, and complete response was also observed in 21% of overall population, regardless of the types of cancers [48]. Moreover, they performed sequencing of T-cell receptor CDR3 regions (TCR seq) on tumors from responders and found that intra-tumoral neoantigen-specific T cells were clonally expanded in peripheral blood [48]. Strikingly, these mutant neoantigen-specific T cell clones were often undetectable in the periphery before administration of pembrolizumab, but these were rapidly expanded after pembrolizumab treatment in responding patients [48].

In this context, genomic instability is one of the genomic hallmarks of IDC-P tumors; therefore, it could make them sensitive to respond well to ICB. Recently, the KEYNOTE-028 study showed that objective response rate was up to 17.4% after pembrolizumab treatment in patients with heavily pretreated, advanced PD-L1-positive PCa [49]. Of note, pembrolizumab monotherapy resulted in durable treatment response (median duration of response = 13.5 months) [49]. The KEYNOTE-199 study also demonstrated antitumor activity (disease control rate of up to 32%), and durable treatment response (median duration of response was not reached in PD-L1-positive disease) in a larger mCRPC population treated by docetaxel and one or more ARAT agents [50]. Currently, the KEYNOTE-921 study is ongoing as a phase 3 trial of pembrolizumab plus docetaxel and prednisone or prednisolone versus placebo plus docetaxel in patients with mCRPC progressing after ARAT therapies [51]. Therefore, there is a clear need for further studies for evaluating the therapeutic role of ICB therapy, such as pembrolizumab monotherapy or combination therapy, in patients with IDC-P components with higher genomic instability as a novel treatment option.

Tumors with deleterious mutations in DNA repair genes, including the HRR pathway, respond well to poly (adenosine diphosphate–ribose) polymerase (PARP) inhibitors, such as olaparib, rucaparib, and niraparib [52,53]. The clinical efficacy of PARP inhibitors is based on the theory of synthetic lethality, where either PARP inhibition or HRR deficiency alone is not lethal, but their combination leads to tumor-specific cell death [54]. One of the earliest studies on the efficacy of PARP inhibitors in prostate cancer was the phase 2 TOPARP-A trial, which shows the feasibility of PARP inhibitors in mCRPC [55]. Notably, this study performed whole-exome sequencing and RNA sequencing using fresh-frozen biopsy tissues before treatment [55]. Among patients with aberrations in DNA damage repair (DDR) genes, 88% responded to olaparib treatment and showed significantly longer PFS and OS than those without DDR gene mutations [55]. More importantly, the PROfound trial, which was a prospective, biomarker-selected, phase 3 study, enrolled patients with mCRPC and disease progression after receiving ARAT agents and showed that median radiographic PFS and OS were significantly longer in the olaparib treatment arm than in the control arm (7.4 months vs. 3.6 months and 18.5 months vs. 15.1 months, respectively), particularly in the cohort with multiple loss-of-function alterations in the HRR pathway [56]. Although these promising results have not been validated in patients with IDC-P histology and concurrent DDR gene mutations, we believe that PARP inhibitors have great potential to overcome the intrinsic resistance to ADT and other conventional therapies for IDC-P-containing prostate cancer with these mutations.

## 4. Patient-Derived Models of IDC-P Tumor for Genomic Studies

Currently, a majority of studies on genomic features of IDC-P used frozen or formalin-fixed tissues derived from patients. However, it is challenging to obtain high-quality samples sufficient for genomic analysis. Patient-derived preclinical models have recently been highlighted as a promising way to overcome this difficulty, because of high take rate of patient-derived samples that are established for a single generation [57]. Thus, patient-derived ex vivo models, such as patient-derived xenografts (PDXs) or tumor organoid (TO), can be useful for establishing rare histological subtypes of tumor [57].

Risbridger et al. [11] generated PDXs model by using fresh radical prostatectomy tissue from three germline *BRCA2* pathogenic mutation carriers and one *BRCA2* wild-type patient. For assessing adenocarcinoma and IDC-P, they finally used a total of 44 PDXs samples from four patients. Porter et al. [58] also established PDXs from seven patients with high-risk PCa to evaluate the treatment response of IDC-P tumor to ADT. Notably, IDC tumor in PDXs maintained its morphological features, such as a cribriform, as well as molecular features, including *AMACR*- and *ERG*-positive luminal cells and *p63*-positive basal cells. Therefore, the PDX model has a great advantage to study distinct pathologies of IDC-P tumors because it could maintain the growth pattern and complex composition of the original tumor, including cribriform morphology and preservation of the basal cell layer. Additionally, IDC-P tumors in PDXs may take up a similar volume to adjacent invasive adenocarcinoma with a substantial tumor burden.

Patient-derived TO is an alternative model to recapitulate the morphologic features and genomic landscape of parent tumors. For instance, Gao et al. [59] established in vitro models of PCa derived from metastatic tumors and circulating tumor cells, and they revealed that these organoid lines recapitulated copy number signatures of original PCa, such as *TMPRSS2–ERG* fusion, *SPOP* mutation, *PTEN* loss, and *CHD1* loss, as well as genomic alterations frequently detected in mCRPC including *TP53, PIK3R1,* and *FOXA1*. Recently, Karkampouna and colleagues generated PCa patient-derived tumor organoids from treatment-naïve metastatic tissues [60]. These TOs showed budding acinar and adenocarcinoma-like architectures, with dominant expression of luminal markers (CK8, PSA, and AR) and less abundant expression of basal markers (CD49f/ITGA6, KRT5, KRT6) [60]. Moreover, they demonstrated that transcriptomic and genomic profiles of tumor organoids and parent tissues were highly similar [60]. In this regard, the patient-derived TO model can allow a better understanding of the complexity of tumor initiation and progression, in addition to being a promising in vitro tool for studying the genomic features of IDC-P tumor. Table 2 describes the advantages and limitations of both PDX and TO models.

## 5. Limitations and Future Perspectives

Despite the growing understanding of molecular features of IDC-P, there are still limitations that need to be addressed by further genomic studies. For example, genomic analyses of IDC-P were usually performed using the microdissection technique [57]. Because this may be laborious to do for larger populations, most genomic data of IDC-P have been derived from relatively small cohorts [57]. In this context, there are some discrepancies in the prevalence of various genomic alterations between different studies. More importantly, current transcriptomic data of IDC-P tumor have been obtained from bulk RNA sequencing, whereby the precise expression profiling of diverse cells, including malignant, immune, and stromal cells, is mostly masked [61]. Single-cell RNA sequencing could precisely measure the gene expression levels of individual cells in a tumor. Thus, single-cell genomic technology is regarded as a powerful tool for comprehensive profiling of human cancers at the resolution of individual cells [61]. We believe that single-cell genomics, particularly single-cell RNA sequencing, should be rigorously performed in IDC-P tumors to decipher more accurate tumor biology, such as aggressive tumor growth, early recurrence, and resistance to therapies.

Additionally, our study is a subjective selection and narrative review of previous reports the authors think are relevant to the topic of genomic features with clinical implication for IDC-P tumors. Thus, the literature review of the current study does not follow a specific methodology or guidelines of systematic review; therefore, this may have caused selection bias in analyzing the current literature on this topic.

## 6. Conclusions

Overall, the current evidence indicates that the aggressive behavior of prostate cancer with IDC-P histology can be partially explained by the high prevalence of LOH in tumor suppressor genes, increased percentage of genomic instability, and high frequency of deleterious defects in DDR genes. Although these molecular hallmarks of IDC-P tumors provide more insights into their optimal therapeutic strategies, information on putative therapeutic targets is still lacking. Further genomic characterization and a better understanding of the fundamental molecular mechanisms of prostate cancer harboring IDC-P histology could be translated into the development of actionable targets for this unique and aggressive type of prostate cancer.

## Figures and Tables

**Table 1:** Histopathological, clinical and genomic characteristics of intraductal carcinoma of the prostate (IDC-P).

**Microscopic features** [7]	• lumen-spanning, expansile growth of atypical cells
	• solid, dense/loose cribriform, micropapillary growth pattern
	• cuboidal or low columnar cells
	• increased mitosis
	• marked nuclear pleomorphism
	• at least, partially preserved basal cell layer
**Coexisting** **lesions** [1,2,7]	• typically, high-grade invasive adenocarcinoma
	• rarely, Gleason grade group 1 or benign acini
**Pathologic** **features** [1,2,7]	• high Gleason grade group
	• larger tumor volume
	• more advanced pathologic stage
	• more extraprostatic extension and lymph node metastasis
**Clinical** **features**	• earlier biochemical recurrence [4,8,9]
	• higher distant metastasis rate [10]
	• poor survival outcomes [8,11]
**Genomic** **features**	• frequent *TMPRSS2–ERG* fusion, loss of *PTEN, RB1*, and *TP53* [12,13,14,15]
	• increased genomic instability (percentage of genome alteration, PGA) [10,16]
	• frequent loss of heterozygosity [13]
	• “nimbosus” phenomenon (higher PGA, hypoxia, higher *SChLAP1*) [17,18,19]
	• frequent mutations of DNA damage repair pathway genes [20,21,22,23]
	• enrichments in MAPK/PI3K pathway genes (isolated IDC-P) [24]
**Systemic** **therapies**	• heterogenous response to androgen-deprivation therapy [25,26,27]
	• AR axis targeting agents > docetaxel [28,29]
	• anti-PD1, PARP inhibitor as promising therapies

**Table 2:** Comparison of advantages and disadvantages of patient-derived xenograft (PDX) and tumor organoid (TO) models.

Models	Advantages	Disadvantages
**Patient-derived** **xenograft (PDXs)**	• Preserves tumor heterogeneity	• Labor-intensive and time-consuming
	• Retains genomic features	• High cost
	• Contains various type of cells in tumor microenvironment	• Use of immune compromised mouse
	• High take rate (~90%)	• Gaps between different species (mouse and human)
	• Can be applied to metastasis model	
	• Biobanking	
**Tumor organoid**	• Preserves tumor heterogeneity	• Low take rate (~30%)
	• Retains genomic features	• Contains only epithelial cells
	• Rapid generation	• No tumor microenvironment
	• Appropriate for high-throughput screening	• Limited passages
	• Can be used for PDX model	• Not evaluable in metastatic disease
	• Biobanking	
